# AKI-to-CKD transition is a potential mechanism for non-albuminuric diabetic kidney disease

**DOI:** 10.12703/r/11-21

**Published:** 2022-07-28

**Authors:** Kyung Lee, John Cijiang He

**Affiliations:** 1 Department of Medicine, Division of Nephrology, Icahn School of Medicine at Mount Sinai, One Gustave L. Levy Place, New York, NY 10029, USA; 2 Renal Section, James J. Peters Veterans Affair Medical Center, 130 W. Kingsbridge Road, Bronx, NY, 10468, USA

**Keywords:** diabetic kidney disease, albuminuria, renal tubular epithelial cells, acute kidney injury, chronic kidney disease, RTN1A, endoplasmic reticulum, mitochondria

## Abstract

Although albuminuria development is considered the natural course of diabetic kidney disease (DKD), increasing evidence indicate that the disease can present as non-albuminuric DKD (NA-DKD), characterized by prominent tubulointerstitial injury and fibrosis without obvious glomerulopathy. However, the pathogenic mechanisms underlying NA-DKD remain unclear. As diabetic patients are more susceptible to acute kidney injury (AKI), and the maladaptive repair of kidney tubules following AKI occurs more frequently in diabetic than non-diabetic patients, the enhanced AKI-to-CKD transition may be a significant contributor of NA-DKD. Recent studies indicate that endoplasmic reticulum (ER) stress is a key pathogenic driver of AKI-to-CKD transition, and that the tubular expression of ER-resident protein reticulon 1A (RTN1A) correlates with human DKD progression and AKI-to-CKD transition. Experimental studies showed that RTN1A indeed mediates tubular cell injury and AKI-to-CKD transition in diabetic mice via concomitant activation of ER stress and mitochondrial dysfunction as a mediator of ER-mitochondrial crosstalk. Further understanding of the pathogenesis of tubular injury in DKD will help us to develop sensitive and specific biomarkers or diagnostic tools to distinguish between injury-related AKI, pre-renal AKI from hemodynamic changes, and the progression of DKD in order to better manage patients with DKD.

## Introduction

Diabetic kidney disease (DKD), also referred to as diabetic nephropathy, is a common complication of diabetes associated with high morbidity and mortality. DKD has been classically defined by glomerular lesions, characterized by glomerular basement membrane (GBM) thickening, mesangial expansion, and podocyte loss at the early stage, diffuse nodular glomerulosclerosis, afferent and efferent arteriolar hyalinosis, and tubulointerstitial fibrosis with inflammation at the later stage of the disease^1^. DKD is clinically diagnosed by the presence of persistent albuminuria and/or reduced estimated glomerular filtration rate (eGFR). Pathologically, DKD could be classified into three categories: (1) classic diabetic glomerulopathy as described above, (2) predominant vascular and interstitial changes with relatively normal glomerular structure (with mild GBM thickening and mesangial expansion), and (3) non-diabetic glomerular disease superimposed on diabetes (without structural changes in GBM or mesangium)^2^. Clinically, patients in the second category often have an early GFR decline without significant albuminuria, referred to as non-albuminuric DKD (NA-DKD), and increasing evidence indicates that some NA-DKD patients, nevertheless, progress to end-stage kidney disease (ESKD)^3,4^. Indeed, epidemiological studies suggest that about 13% of NA-DKD patients have a declining eGFR, representing nearly 50% of patients with DKD^5^. However, the pathogenesis of NA-DKD remains unclear. Here we will discuss the current evidence highlighting the acute kidney injury (AKI)-to-CKD transition as a potential mechanism in NA-DKD development in patients with diabetes.

## ER stress-induced tubular injury as a key culprit in AKI-to-CKD transition in diabetes

A recent large observational study of 16,700 patients suggests that patients with diabetes have significantly higher rates of AKI compared with patients without diabetes^6^. As summarized in a recent review in 2018^7^, the high susceptibility of AKI in patients with diabetes could be due to three major mechanisms: (1) increased systemic inflammation in diabetes, (2) diabetes-associated vascular disease, and (3) increased susceptibility of kidney tubular cells to injury. In animal models, AKI is more severe in diabetic mice than in control mice, and hyperglycemia-induced activation of the p53 pathway and mitochondrial dysfunction are major contributors to the high susceptibility of AKI in diabetes^8^.

Both epidemiological and experimental studies suggest that patients with AKI can progress to CKD^9,10^, depending on the severity and frequency of AKI^11^. After AKI, surviving tubular epithelial cells can undergo dedifferentiation for proliferation to start the repair process. A proper repair response can restore tubular epithelial integrity and function, whereas maladaptive repair results in the development of CKD^12,13^. In patients with diabetes, hyperglycemia and diabetic milieu could contribute to maladaptive repair of injured tubular cells following AKI^7,14^. Moreover, patients with diabetes often suffer multiple episodes of AKI due to vascular changes, endothelial cell injury, toxicity associated with medications, and multiple surgeries, while some episodes of mild AKI may go undetected. Thus, episodes of AKI in patients with diabetes are likely responsible for DKD transition. It is also likely that AKI-to-CKD transition is responsible for the decline of eGFR in patients with NA-DKD, which is characterized mostly by tubulointerstitial injury and fibrosis.

Tubular cell injury also contributes to the progression of DKD in general. It is well recognized that tubular cell injury occurs at early DKD prior to the onset of microalbuminuria^15^, even though the morphological changes of tubules are poorly characterized at the early stage^16^. It has been shown that renal function correlates more with the degree of tubulointerstitial injury than that of the glomerular lesions in patients with DKD^1^. A large body of evidence indicates that hyperglycemia and advanced glycation end products in diabetes contribute to tubular epithelial cell injury^17–19^. Proteinuria, caused by diabetic glomerulopathy, also contributes to tubular cell injury and fibrosis development^20^. In addition to these, episodes of AKI and subsequent maladaptive repair of tubular cells are likely the major culprits in accelerating the progression of DKD. Indeed, many patients with DKD progress to ESKD without a transition from macroalbuminuria to overt proteinuria, indicating again the importance of tubular cell injury independent of glomerular injury.

In 2015, by analyzing the transcriptomic datasets in animal models with different rates of disease progression, we identified the reticulon 1 gene (*Rtn1*), which encodes reticulon 1A (RTN1A), as a risk factor for progression of DKD^21^. Reticulons localize primarily to the endoplasmic reticulum (ER) membrane as ER-shaping proteins. RTN1A expression is markedly increased in the tubular cells of diabetic kidneys, and its expression is inversely correlated with eGFR in patients with DKD^21^. Three intronic single-nucleotide polymorphisms of RTN1 are associated with diabetic patients with ESKD^22^. Notably, RTN1A expression also increases in the kidney of patients with AKI. The tubular expression of RTN1A correlates with the severity of AKI, such that higher expression of tubular RTN1A expression is observed in kidneys of patients with progressive AKI (indicative of maladaptive repair) as compared with those of non-progressive AKI (indicative of normal repair)^23^, suggesting a pivotal role of RTN1A in AKI-to-CKD transition.

Using mouse models, we indeed confirmed that RTN1A worsened tubular cell injury in AKI and promoted AKI-to-CKD transition^23^. In diabetic mice, the induction of RTN1A expression, specifically in tubular cells, markedly reduced renal function while enhancing significant tubulointerstitial fibrosis, both of which are not typically observed in mouse models of DKD^24^. Thus, together with the observations in human AKI patients, the experimental studies implicate a role of RTN1A in AKI-to-CKD transition in diabetes.

**Figure 1.**
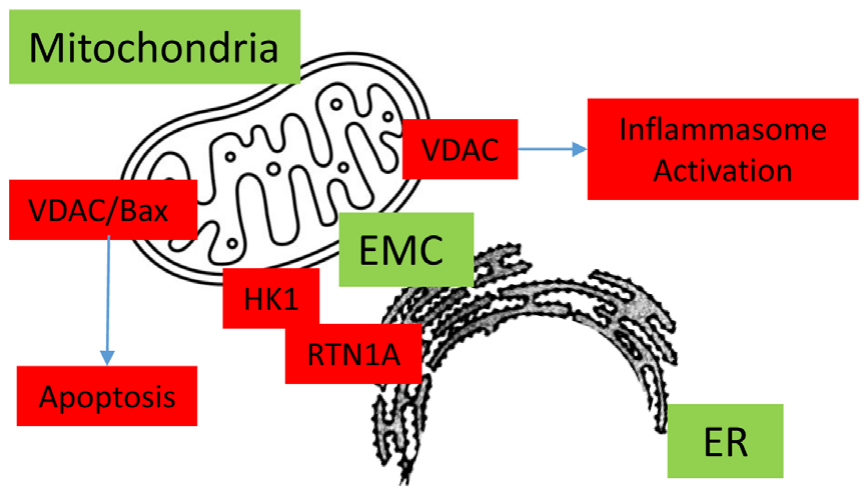
RTN1A injury kidney cells via activation of ER-mitochondrial contact. RTN1A-HK1 interaction disrupts HK1-VDAC1 interaction, thereby releasing VDAC1 to induce inflammasome- and BCL2-mediated apoptosis. EMC, endoplasmic reticulum-mitochondrial contact; ER, endoplasmic reticulum; HK1, hexokinase-1; RTN1A, reticulon 1A; VDAC, voltage-dependent anion channel.

Mechanistically, RTN1A enhances ER stress and apoptosis in kidney tubular epithelial cells through interaction with PERK, leading to persistent PERK dimerization and activation^21^. Interestingly, many RTN1A-interacting proteins identified by mass spectrometry were enriched in outer membrane mitochondrial proteins^24^, suggesting that RTN1A may regulate both ER stress and mitochondrial dysfunction through acting on the ER-mitochondrial contact (EMC) sites. Consistent with this, a 2017 study also demonstrates a large amount of RTN1A in the EMCs, also known as mitochondria-associated ER membranes (MAMs)^25^. Amounting evidence indicates that the alterations in EMCs have pleiotropic effects on a variety of intracellular events, including mitochondrial dysfunction, apoptosis, and autophagy, which are known to be involved in the pathogenesis of DKD^26–29^. Interestingly, overexpression of RTN1A results in closer ER and mitochondrial contacts. Moreover, ER-bound RTN1A altered the interactions between mitochondrial hexokinase-1 (HK1) and voltage-dependent anion channel-1 (VDAC1), thereby resulting in apoptosis and inflammasome activation, ultimately leading to tubular cell injury and loss (Figure 1). These findings illustrate a novel mechanism of tubular cell injury and provide a molecular basis for AKI-to-CKD transition in patients with diabetes.

Clinically, AKI in patients with DKD is often unrecognized or missed during routine follow-up visits. With the current treatments including renin-angiotensin system blockade, sodium glucose transporter 2 inhibitors, and diuretics, serum creatinine often fluctuates in patients with DKD. Therefore, it is difficult for clinicians to distinguish between injury-related AKI, pre-renal AKI due to drug-induced hemodynamic changes, and natural progression of DKD when patients have increased serum creatinine. Tubular cell injury markers such as kidney injury molecule (KIM)-1 might be helpful for differential diagnosis in these patients. A recent study in 2022 demonstrated the clinical utility of a diagnostic tool, KidneyIntelX, that incorporates three biomarkers (soluble tumor necrosis factor receptor-1 and -2, and KIM-1) and clinical variables in improved prediction of kidney outcomes over KDIGO (Kidney Disease: Improving Global Outcomes) and clinical models in individuals with early stages of DKD. Thus, it is plausible that such approaches may help distinguish between drug-induced pre-renal and injury-related AKI in these patients with diabetes. We are in the process of developing RTN1A as a potential novel tubular cell injury marker, which could improve the prediction of tubular cell injury. The distinction between natural progression of DKD, injury-related AKI, and pre-renal events from drug-induced hemodynamic changes could help clinicians to better manage these patients with DKD.

In summary, patients with diabetes are more susceptible to AKI and often have multiple episodes of AKI. Maladaptive AKI occurs more often in patients with diabetes, particularly in those with DKD. AKI-to-CKD transition may contribute to the progression of DKD and the pathogenesis of NA-DKD in patients with diabetes. Our study suggests that RTN1A might be one of the mechanisms mediating the AKI-to-CKD transition in patients with diabetes. The distinction between natural DKD progression, injury-related AKI, and pre-renal AKI from drug-induced hemodynamic changes by using newly developed diagnostic tools could help clinicians to better manage patients with DKD.
